# General practitioners’ attitudes towards early diagnosis of dementia: a cross-sectional survey

**DOI:** 10.1186/s12875-019-0956-1

**Published:** 2019-05-20

**Authors:** Stéphanie Giezendanner, Andreas U. Monsch, Reto W. Kressig, Yolanda Mueller, Sven Streit, Stefan Essig, Andreas Zeller, Klaus Bally

**Affiliations:** 1 Centre for Primary Health Care, University of Basel, Basel, Basel, Switzerland; 2 Memory Clinic, University Department of Geriatric Medicine FELIX PLATTER, Faculty of Psychology, University of Basel, Basel, Switzerland; 3 University Department of Geriatric Medicine FELIX PLATTER, Faculty of Medicine, University of Basel, Basel, Switzerland; 4 Center for Primary Care and Public Health (Unisanté), University of Lausanne, Lausanne, Switzerland; 5 Institute of Primary Health Care (BIHAM), University of Bern, Bern, Switzerland; 6 Institute of Primary and Community Care Lucerne, Lucerne, Switzerland

**Keywords:** Attitudes, Dementia, Primary care, Early diagnosis, Barriers

## Abstract

**Background:**

Dementia is often underdiagnosed in general practice, which may be based on general practitioners’ (GPs’) knowledge and emotional factors as well as external problems. This study aimed to describe GPs’ attitudes toward early diagnosis of dementia.

**Methods:**

Cross-sectional postal survey in Switzerland in 2017. Members of the Swiss Association of General Practitioners (*N* = 4460) were asked to participate in the survey. The questionnaire assessed attitudes, enablers and barriers to early dementia diagnosis and post-diagnostic intervention strategies. Exploratory factor analysis and linear regression were used.

**Results:**

The survey response rate was 21%. 85% of GPs agreed with enablers of early dementia recognition (e.g. “Plan for the future, organize support and care”, “Minimize the strain and insecurity of patients and their informal family caregivers”). On the other hand, 15% of respondents perceived barriers towards early dementia recognition (e.g. “Time constraints in carrying out the necessary procedures to diagnose dementia”). GPs who were more likely to agree with barriers would less often counsel family members (β = − 0.05, 95% CI = − 0.09 - -0.02) or test fitness to drive (β = − 0.05, 95% CI = − 0.09 - -0.02), and more often choose a watchful waiting strategy (β = 0.05, 95% CI = 0.02–0.09).

**Conclusions:**

The attitude of the majority of GPs is not characterized by diagnostic and therapeutic nihilism. However, negative attitudes were associated with sub-optimal management after the diagnosis. Thus, health systems are required to critically examine the use of available resources allowing GPs to look after patients and their relatives in a holistic way.

**Electronic supplementary material:**

The online version of this article (10.1186/s12875-019-0956-1) contains supplementary material, which is available to authorized users.

## Background

A central aim of strategies on dementia is to enable high quality, low-threshold and continuing provision of health-care services for individuals with dementia [1–3]. The diagnosis of dementia is an important step for access to care and support, and the prevention of stress or crises for carers; and it is advantageous for individuals and their families in how they cope with the prognosis [4].

General practitioners (GPs) have regular contact with the majority of the elderly population and therefore play a pivotal role in the assessment of incipient cognitive decline and dementia. Nevertheless, a high rate of underdiagnosis of dementia has been reported in primary care [3, 5–8]. A systematic literature review found important barriers to early diagnosis of dementia in primary care, including lack of support, time and financial constraints, stigma, diagnostic uncertainty, and GP’s fear that disclosure could damage the doctor-patient relationship [9]. Evidence also revealed other important themes such as delayed presentation and therapeutic nihilism [9].

In contrast, the benefits of early recognition of dementia include receiving early access to treatment, appropriate information, advice and support to improve the quality of life of patients, caregivers, and relatives [10–12]. In particular, patients can be involved in decision-making processes and have the possibility of independent planning for the future (e.g. living will or advanced care plan) [4]. In addition, timely measures can be taken in order to prevent endangering themselves or others (e.g. driving a car, or professional responsibility at work). Early GP interventions have been shown to help caregivers in anticipating and accepting the future care role and transitions, with the increased possibility that caregivers can still involve the patient in the decision making process [12]. Further, dementia care management has been shown to decrease the burden on and associated health impairments of caregivers [13]. Further, people with dementia and their caregivers have recently highlighted the need for GPs to engage in counselling and in signposting of local services [14].

To inform efforts to increase the quality of health-care services for individuals with dementia it is essential to better understand the drivers of GPs’ attitudes towards dementia, including underlying enablers and barriers in the early recognition of dementia across GP and practice characteristics. This may help to identify whether there is a need for tailored education, training or support. Prior studies investigating GPs’ attitudes towards early dementia recognition have found an association with age [15], location of practice [16], gender, and professional experience [17]. Further, GPs’ decisions to diagnose dementia have been shown to be influenced by their own beliefs about dementia and the efficacy of treatment [18]. However, these studies have tended to focus on a limited set of attitudes [15, 19, 20] and have rarely examined the association with GPs’ current management of dementia patients [21, 22].

In Switzerland, the primary care system is mostly based on fee for service. Even if a GP provides a treatment the patient does not actually need, the GP will still be compensated for it. In retrospect, it is practically impossible to prove that a medical service would, in fact, have been unnecessary. Currently there are no binding guidelines relating to medical treatment that help determine which measures should be deemed necessary and expedient for a certain medical condition and which ones can no longer be considered appropriate. Consequently, this means that there are no formal restrictions on the comprehensive diagnosis and treatment of people with suspected dementia.

The main question of this study was whether certain attitudes as well as enablers and barriers to dementia recognition are reflected in the way GPs manage patients with confirmed or suspected dementia, i.e. their professional approach. The latter is exemplified by the average stage of dementia at the point at which a patient is first diagnosed, as well as the GP’s choice of different treatment options when assessing a case vignette of mild dementia. Building on the existing literature, we sought to investigate a broad range of attitudes as well as enablers and barriers to early recognition of dementia, using factor analysis to group them into underlying themes. We then explored the interrelation between attitude themes towards early recognition of dementia and professional approach to the disease and its management.

## Methods

The project was conducted by the Centre for Primary Health Care at the University of Basel, and was supported by all academic institutes of general practice at Swiss universities.

The present cross-sectional postal survey was designed to test attitudes to the early recognition of dementia and dementia care [15, 16, 19, 23–25] as well as barriers and enablers [9, 26–29]. GP’s were also asked to indicate at which stage most of their patients received the first diagnosis. The stage of dementia at the point of first diagnosis [3] comprised MCI, mild, moderate and severe dementia. The definitions of the stages were explained in the questionnaire and were based on a classification from a national consensus [30] (see Supplementary Information S 1).***.*** The attitude items were based on two previous questionnaires about GPs’ attitudes on dementia [15, 19] and comprised statements such as “The early recognition of dementia usually serves the welfare of the patient/patient’s relative” or “Managing dementia is more often frustrating than rewarding”. Barriers and enablers of early dementia were also based on previous findings from literature [9, 24–27] and contained statements such as “With a timely diagnosis GPs/patients may take actions to improve disease outcome, delay institutionalization, reduce dangerous and difficult situations etc.). For all questionnaire items see Supplementary Information S 3. The questionnaire further assessed the management approach after diagnosis of a hypothetical case of mild dementia. The vignette comprised the question “What measures would you take if a patient was diagnosed with an early stage Alzheimer’s disease? (MMSE of 24 and the need of some assistance in activities of daily living)”. The items of these post-diagnostic intervention strategies have been presented in detail elsewhere [31]. All items were assessed using a five-point Likert scale, except the stage of dementia at the point of first diagnosis (MCI, mild, moderate or late stage), and the demographic characteristics of the GP. Content validity was pre-tested among a small group (*n* = 7) of GPs for readability and acceptability. The initial questionnaire was developed in German and two independent translations in French and Italian were made by professional translators. The meaning and the appropriateness of the translated items were assessed by the study team.

The survey was sent by mail to all GP members of the Swiss Association of General Practitioners and Paediatricians (*n* = 4460) in August 2017. A reminder was sent to all members by e-mail 1 month later. Responses were collected anonymously.

We used descriptive statistics to summarize physician and practice characteristics. To identify underlying themes among GP attitudes for or against early dementia recognition, we performed exploratory factor analysis (EFA) with *n* = 2 factors [32] (see Supplementary Information S 6, S 7, S 8). A summary score was created for each factor, ranging from 1 to 5 (see S 6). We determined the association between the summary scores and the following predictors; respondents’ demographic and practice characteristics, average stage of dementia diagnosis, and management approach, using univariable and multivariable linear regression models (for each summary score separately). Multivariable models were adjusted for age, workload, practice location and the estimate of the percentage of patients over the age of 70. Missing data were not imputed and regression analyses were performed on complete cases. *P* values of < 0.05 were considered statistically significant. All data analyses were conducted using R version 3.4.3 [33].

## Results

### Sample

Of the 4460 GPs initially contacted, 306 were either no longer practising as a GP, or the letter was undeliverable; this left a sample of 4154 GPs. A total of 882 (21%) returned the questionnaire. The respondent GPs were 55.8 (SD = 8.86) years old on average, and 70% were male (see Additional file 1: Table S2 for summary statistics of demographic characteristics). The responding GPs did not differ from the contacted GPs in terms of gender (χ^2^ = 0.25, df = 1, *p*-value = 0.617). However, responding GPs did differ from the contacted GPs in terms of language region (χ^2^ = 6.1, df = 2, *p* = 0.046) as there were more German-speaking (78%) respondents compared to the contacted sample (75%). Further, on average the respondents were 1 year younger than the whole contacted sample (55.8 vs. 56.7 years, t = − 2.77, *p* = 0.006).

### General attitude towards early recognition of dementia and care for patients with dementia

**Fig. 1:**
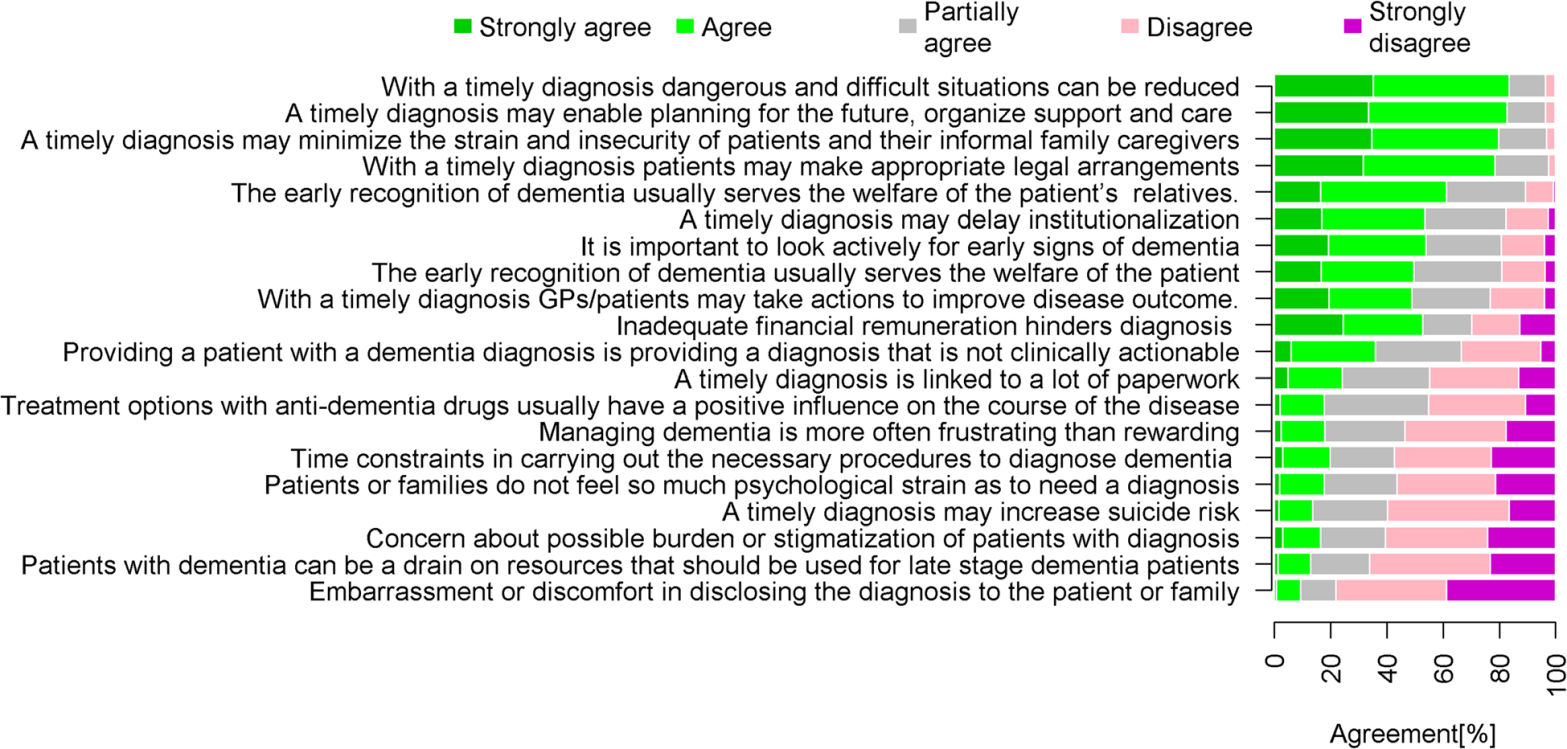
Ranked attitudes towards early dementia diagnosis. Attitudes were ranked according to their agreement (strongly agree and agree)

Figure 1 and Additional file 1: Table S3 show a general attitude that is positive, characterized by the feeling that early diagnosis was beneficial. In particular, more than half of responding GPs perceived a benefit for the welfare of the patient’s relatives (61% agreed/strongly agreed), while half of them saw a benefit to the patients themselves, and did not think the management of dementia was frustrating. Negative or nihilistic attitudes were less prevalent; 18% stated the management of dementia to be frustrating, 33% agreed that providing a patient with a dementia diagnosis is providing a diagnosis that is not clinically actionable, and 45% did not feel that current treatment options (e.g. anti-dementia drugs) had a positive effect on the course of the disease. The majority of GPs agreed with most enablers to timely diagnosis (except “With a timely diagnosis GPs/patients may take action to improve disease outcome”) and disagreed with most barriers (except “Inadequate financial remuneration hinders diagnosis”). Most GPs (62.5%) reported an average stage of “mild dementia” at the point of first diagnosis, 31.2% of respondents reported average stage of “mild cognitive impairment” at the point of first diagnosis, and 6.2% of GPs reported “moderate stage of dementia” at the point of diagnosis. The frequencies of Likert Scale answers of post-diagnostic intervention strategies are described in Additional file 1: Table S4.

### Exploratory factor analysis

The EFA yielded an 18-item scale with 2 factors explaining 34% of the variance (see Table 1 and Additional file 1: Tables S5, S6, S7 and S8 for more details on factor analysis). The first factor comprised GPs’ enablers to providing early dementia diagnosis, which was supported by 85% of GPs. The second factor comprised barriers to providing early dementia diagnosis, and was supported by 15% of GPs (see Table 1 for details on agreement with a factor). The majority of GPs (73%) exclusively agreed with enablers of early dementia diagnosis, 12% agreed with both enablers and barriers, and 3% exclusively agreed with barriers to early dementia diagnosis.

**Table 1:** Rotated factor loadings and unique variances from principal-component factor analysis

Items “A timely diagnosis …	Factor 1	Factor 2	Uniqueness	Mean scores (SD)
Enablers of early dementia recognition				
It is important to look actively for early signs of dementia	0.69	−0.08	0.51	3.66 (0.58)
The early recognition of dementia usually serves the welfare of the patient	0.72	−0.12	0.46	
The early recognition of dementia usually serves the welfare of the patient’s relatives.	0.47	−0.12	0.77	
The present treatment options with anti-dementia drugs usually have a positive influence on the course of the disease	0.41	0.08	0.83	
With a timely diagnosis GPs/patients may take actions to improve disease outcome.	0.57	0.06	0.67	
A timely diagnosis may delay institutionalization	0.58	0.00	0.67	
With a timely diagnosis dangerous and difficult situations can be reduced	0.66	−0.04	0.56	
A timely diagnosis may enable planning for the future, organize support and care	0.73	−0.11	0.46	
A timely diagnosis may minimize the strain and insecurity of patients and their informal family caregivers	0.68	−0.17	0.51	
With a timely diagnosis patients may make appropriate legal arrangements	0.54	−0.09	0.70	
Barriers to early dementia recognition				
Patients with dementia can be a drain on resources that should be used for late stage dementia patients	−0.40	0.45	0.63	2.47 (0.59)
Concern about possible burden or stigmatization of patients with diagnosis	−0.40	0.54	0.55	
Embarrassment or discomfort in disclosing the diagnosis to the patient or family	−0.05	0.54	0.71	
Time constraints in carrying out the necessary procedures to diagnose dementia	0.01	0.59	0.65	
Inadequate financial remuneration hinders diagnosis	0.30	0.45	0.71	
A timely diagnosis may increase suicide risk	0.05	0.53	0.71	
Patients or families do not feel so much psychological strain as to need a diagnosis	−0.36	0.49	0.63	
A timely diagnosis is linked to a lot of paperwork	0.07	0.59	0.65	
Providing a patient with a dementia diagnosis is providing a diagnosis that is not clinically actionable	−0.23	0.26	0.88	3.03 (1.02)
Managing dementia is more often frustrating than rewarding	−0.10	0.28	0.91	2.49 (1.03)

Most variables of the first factor loaded substantially onto only one factor. From the variables loading positively onto the second factor, 3 variables also loaded negatively onto the first factor. Further, there were two items that did not fit well in the solution (“Managing dementia is more often frustrating than rewarding” and “Providing a patient with a dementia diagnosis is providing a diagnosis that is not clinically actionable”). Mean scores represent the mean agreement in responses for each scale, with 1 indicating a low agreement and 5 the highest possible agreement. Agreement is represented by the percentage of mean scores higher than 3 across responding GPs

### Association between GPs’ attitudes and demographic characteristics or professional approach

The regression results are presented in Table 2. Younger GPs were more likely than older GPs to agree with enablers but also with barriers to early dementia diagnosis. Compared to men, women were more likely to agree with barriers to early dementia diagnosis. GPs who reported a later average stage at first diagnosis (one point increase e.g. from “MCI” to “mild dementia”) also agreed less with enablers of early dementia diagnosis. GPs with higher agreement of enablers of early dementia diagnosis were significantly more likely to take all the proposed measures after a hypothetical case of mild dementia diagnosis, except watchful waiting. GPs who agreed more with barriers to early dementia recognition would counsel family members less often after a diagnosis of mild dementia and would more often adopt a watchful waiting strategy. Further, GPs who agreed more with barriers to early dementia recognition would more often prescribe *Ginkgo biloba* after a diagnosis of mild dementia, and would test fitness to drive less often after a diagnosis of mild dementia.

**Table 2:** Linear regression models of the association between GPs’ attitudes, demographic characteristics, interventions and first diagnosis

	Univariable model				Multivariable model			
	Est	95% CI		*p* value	Est	95% CI		*p* value
Enablers of early dementia diagnosis								
Demographics								
Age (per 10 years)	− 0.07	− 0.12	− 0.03	0.001	− 0.08	− 0.12	− 0.03	0.001
Sex (female vs. male)	0.13	0.05	0.21	0.002	0.10	0.00	0.20	0.055
Workload (per 10 h per week)	0.00	− 0.03	0.02	0.777	0.00	− 0.03	0.02	0.786
Consultations (per 5 per day)	− 0.02	− 0.06	0.02	0.423	− 0.02	− 0.07	0.02	0.335
Region (agglomeration vs. city)	− 0.01	− 0.11	0.08	0.823	− 0.01	− 0.11	0.09	0.851
Region (rural vs. city)	0.06	− 0.03	0.16	0.189	0.07	− 0.02	0.17	0.130
Estimate of the percentage of patients over the age of 70 (per 10%)	0.00	− 0.02	0.02	0.785	0.00	− 0.02	0.02	0.840
Language (French vs. German)	− 0.09	− 0.19	0.01	0.080	− 0.08	− 0.18	0.03	0.145
Language (Italian vs. German)	0.18	0.00	0.37	0.056	0.19	0.00	0.39	0.052
Interventions								
Watchful waiting	− 0.14	− 0.18	− 0.11	< 0.001	− 0.13	− 0.17	− 0.10	< 0.001
Therapy with AChEI or memantine	0.14	0.11	0.17	< 0.001	0.14	0.11	0.18	< 0.001
Prescription of *Ginkgo biloba*	0.04	0.01	0.07	0.012	0.04	0.01	0.07	0.009
Minimize cardiovascular risk	0.07	0.03	0.10	< 0.001	0.07	0.04	0.10	< 0.001
Memory training (groups)	0.10	0.07	0.13	< 0.001	0.10	0.07	0.13	< 0.001
Counselling for family members	0.10	0.06	0.14	< 0.001	0.10	0.06	0.14	< 0.001
Other therapies (music, painting, dancing, coaching)	0.09	0.06	0.12	< 0.001	0.09	0.06	0.13	< 0.001
Prepare advance directives	0.10	0.07	0.13	< 0.001	0.10	0.07	0.13	< 0.001
Test fitness to drive	0.10	0.07	0.13	< 0.001	0.10	0.07	0.13	< 0.001
Counselling for the patient (e.g. AD association)	0.11	0.08	0.14	< 0.001	0.12	0.09	0.15	< 0.001
Diagnosis								
Average point of first diagnosis	− 0.08	− 0.14	− 0.01	0.028	− 0.07	− 0.15	0.00	0.039
Barriers to early dementia diagnosis								
Demographics								
Age (per 10 years)	− 0.07	− 0.11	− 0.02	0.005	− 0.06	− 0.11	− 0.02	0.007
Sex (female vs. male)	0.15	0.07	0.24	0.001	0.13	0.03	0.24	0.010
Workload (per 10 h per week)	− 0.01	− 0.04	0.02	0.490	0.00	− 0.03	0.03	0.945
Consultations (per 5 per day)	0.02	− 0.02	0.06	0.333	0.02	− 0.03	0.07	0.427
Region (agglomeration vs. city)	0.09	− 0.01	0.19	0.079	0.05	− 0.05	0.15	0.320
Region (rural vs. city)	− 0.02	− 0.12	0.07	0.660	− 0.04	− 0.14	0.06	0.423
Estimate of the percentage of patients over the age of 70 (per 10%)	− 0.01	− 0.03	0.02	0.554	− 0.01	− 0.03	0.01	0.372
Language (French vs. German)	− 0.10	− 0.20	0.00	0.060	− 0.09	− 0.20	0.01	0.087
Language (Italian vs. German)	− 0.50	− 0.69	− 0.31	< 0.001	− 0.48	− 0.68	− 0.29	< 0.001
Interventions								
Watchful waiting	0.05	0.02	0.09	0.003	0.05	0.01	0.09	0.007
Therapy with AChEI or memantine	− 0.01	− 0.05	0.02	0.401	− 0.02	− 0.05	0.02	0.408
Prescription of ginkgo biloba	0.07	0.04	0.10	< 0.001	0.06	0.03	0.09	< 0.001
Minimize cardiovascular risk	0.00	− 0.03	0.03	0.946	0.00	− 0.03	0.04	0.850
Memory training (groups)	− 0.02	− 0.05	0.01	0.110	− 0.03	− 0.06	0.01	0.103
Counselling for family members	− 0.05	− 0.09	− 0.02	0.006	− 0.06	− 0.09	− 0.02	0.006
Other therapies (music, painting, dancing, coaching)	− 0.03	− 0.06	0.01	0.116	− 0.03	− 0.06	0.00	0.089
Prepare advance directives	− 0.03	− 0.06	0.00	0.088	− 0.03	− 0.06	0.00	0.064
Test fitness to drive	− 0.05	− 0.09	− 0.02	0.001	− 0.06	− 0.10	− 0.03	< 0.001
Counselling for the patient (e.g. AD association)	0.01	− 0.02	0.05	0.502	0.01	− 0.03	0.04	0.749
Diagnosis								
Average point of first diagnosis	0.06	− 0.01	0.13	0.078	0.07	− 0.01	0.14	0.073

*Est* beta coefficient of linear regression, *CI* confidence interval

For each unit increment of the independent variables (e.g. in the frequency of an intervention from 0 to 25% of cases or for each increment in the average moment of first diagnosis from MCI to mild dementia), the attitude of GPs changed by the indicated estimate on a scale ranging from 1 to 5. The multivariable models were adjusted for age, work (hours per week), practice location (city, agglomeration of the city, countryside), and the estimate of the percentage of patients over the age of 70

Table 2 about here.

## Discussion

The main findings of this research are that 85% of respondents revealed a positive attitude towards early dementia recognition while 15% agreed that there are barriers to early dementia recognition. A minority showed nihilistic attitudes, believing that providing a patient with a dementia diagnosis is providing a diagnosis that is not clinically actionable (33%), or feeling that the present treatment options with anti-dementia drugs had no positive influence on the course of the disease (45%). GPs’ self-reported positive attitudes towards enablers of early dementia recognition were associated with an increase in the number of measures taken after mild dementia diagnosis, and an earlier average point of first diagnosis. GPs reporting more often that there are barriers to early recognition of dementia would less often counsel family members or test fitness to drive. Instead they would more often use the wait and see strategy, or prescribe *Ginkgo biloba*.

The generally positive attitude towards early dementia recognition and dementia care is consistent with recent literature findings indicating that GPs are dedicated to and concerned with caring for their patients with dementia, and that they acknowledge the benefits to patients and their carers of a timely dementia diagnosis at an early stage of the disease [20, 34–37]. Notably, this positive attitude was present although 45% of the respondents held the view that anti-dementia drugs had no positive influence on the clinical course of the disease. Variables loading most on the first factor were associated with long-term planning, taking into account not only physical and mental well-being, but also social aspects. GPs with this understanding are rooted in the thinking of advance care planning [38] and palliative care [39, 40]. All these aspects are crucial in order to educate newly diagnosed patients and their family members about coping strategies and about maintaining independence [41].

The current results are in line with findings from other literature showing the significance of time and financial constraints for diagnostic assessments and post-diagnostic dementia management [9, 20]. The current study showed that GPs agreeing more that there are barriers would more often choose the “wait and see” approach to diagnosis and management, less often test fitness to drive or counsel relatives. Thus, GPs negative attitude may threaten optimal management of persons with dementia increasing the likelihood that dangerous situations, stress or crisis for the patient and relatives would occur. Attitudes based on a “therapeutic nihilism” were not prevalent among current respondents. In fact, only 18% agreed that managing dementia is more often frustrating than rewarding, which is in accordance with recent findings [42]. Nevertheless, 36% of current respondents agreed that providing a patient with a dementia diagnosis is providing a diagnosis that is not clinically actionable, which seems to be more frequent compared with findings from literature (5%) [37]. Time of diagnosis does not solely depend on GP’s, but also is a patient and patients’ caregiver issue. Findings of a focus group study with people with dementia, informal carers and health and social care professionals in eight European countries has shown that the attitudes and beliefs of people with dementia and their carers may have a major impact on the access to formal care, and they often serve as barriers [43]. Formal care was perceived as a threat to the individual independence of people with dementia and was thus avoided as long as possible. Thus, if patients generally present later to GPs, this may compound with GPs therapeutic nihilism.

We have seen that most GPs agreeing that there are barriers to early dementia recognition at the same time agree with the enablers of early dementia recognition. Thus, it is important to motivate and empower those GPs. Political measures involving e.g. monetary incentives appeared to have been effective in closing the gap between recorded and expected prevalence of dementia in primary care [44–46]. Since there are no signs of significant progress in dementia therapy in the foreseeable future, we suggest that GP training should combine positive attitudes towards dementia care with a special focus on a holistic advance care planning approach to care for patients with dementia. This should include their caregivers in order to prevent crises, and should involve the patient in the decision-making process [38, 47]. Hopefully, the proposed approach will provide GPs who will lose or have lost their belief in the efficacy of anti-dementia drugs with a viable alternative. Recent surveys in Europe indicate that positive attitudes towards early dementia recognition and care are becoming more prevalent [37, 45].

The limited response rate of 21% may compromise the generalisability of the results, although a 20–30% participation rate is very common in population-based surveys in primary care [48–50]. In particular in view of the low priority with which dementia is regarded by GPs [51], we anticipated a low participation rate. The low response rate might lead to selection bias, e.g. an over-representation of GPs who were interested in the topic of dementia, thereby overestimating the positive attitude to early dementia recognition. Considering demographic characteristics, the respondents differed minimally from the total sample in terms of gender (70% vs 69% males), age and language region. Respondents were in average 1 year younger than in the total sample (55.8 vs 56.7 years) and there were more German-speaking respondents than in the total sample (79% vs 75%) (see Supplementary Information S 2). Another limitation is that the ratings represent the GPs’ perceptions, and their judgements may lack objectivity.

## Conclusions

In the light of recent disappointing results of both systematic reviews of existing anti-dementia drugs [52] and clinical trials with newer anti-dementia drugs [53, 54], GPs estimate the effectiveness of available anti-dementia drugs on the course of the disease to be low. However, the attitude of the majority of GPs is not characterized by diagnostic or therapeutic nihilism. In particular, young GPs favour early diagnosis of dementia for timely advance care planning, prevention of dangerous situations, and timely counsel and support for relatives and patients with the aim of influencing disease outcome and delaying institutionalization. These measures are time consuming and costly, which could jeopardize the success of this desirable approach. Health systems are therefore required to critically scrutinize the use of available resources. It is essential to support training, care and research that will allow GPs to look after patients and their relatives in a holistic way, so that they can manage and live with the diagnosis of dementia.

## Additional file

Additional file 1: **Table S1.** Stages of dementia at the point of first diagnosis used in the questionnaire. **Table S2.** Demographic, regional and professional characteristics of the respondent GPs. **Table S3.** Frequency of agreement with attitudes towards timely diagnosis (*N* = 882). **Table S4.** Frequency and summary statistics of measures taken after the diagnosis of mild dementia. **Table S5.** Supplementary information on exploratory factor analysis. **Table S6.** Figure: Ranked attitudes regarding dementia recognition and care. Attitudes were ranked according to GPs’ agreement. **Table S7.** Supplementary measures for the quality of the scales. **Table S8.** Summary table for subscales of attitudes of agreement with attitudes towards timely diagnosis. (DOCX 205 kb)

## Abbreviations

CI: Confidence interval
DF: Degrees of freedom
EFA: Exploratory factor analysis
GP: General practitioner
MCI: Mild cognitive impairment
MMSE: Mini-Mental Status Examination
SD: Standard deviation

## Authors’ information

SG: Degree in Psychology, PhD in Health Sciences (Neurosciences). Post-doctoral fellow at the Centre for Primary Health Care, University of Basel.

## References

[1] Monsch AU, Büla C, Hermelink M, Kressig RW, Martensson B, Mosimann U (2012). Konsensus 2012 zur Diagnostik und Therapie von Demenzkranken in der Schweiz. Praxis.

[2] Federal Office of Public Health, Swiss Conference of Cantonal Health Directors. Nationale Demenzstrategie 2014–2017. 2013. https://www.bag.admin.ch/bag/en/home/strategie-und-politik/nationale-gesundheitsstrategien/nationale-demenzstrategie.html.

[3] ALCOVE Project. The European joint action on dementia. Synthesis Report 2013. ALzheimer COoperative Valuation in Europe (ALCOVE) 2013 [Available from: https://ec.europa.eu/health/sites/health/files/major_chronic_diseases/docs/2014_implreport_alzheimer_dementias_en.pdf.

[4] Woods R, Moniz-Cook E, Iliffe S, Campion P, Vernooij-Dassen M, Zanetti O (2003). Dementia: issues in early recognition and intervention in primary care. J R Soc Med.

[5] Connolly A, Gaehl E, Martin H, Morris J, Purandare N (2011). Underdiagnosis of dementia in primary care: variations in the observed prevalence and comparisons to the expected prevalence. Aging Ment Health.

[6] Eichler T, Thyrian JR, Hertel J, Kohler L, Wucherer D, Dreier A (2014). Rates of formal diagnosis in people screened positive for dementia in primary care: results of the DelpHi-trial. J Alzheimers Dis.

[7] Prince M, Bryce R, Ferri C. World Alzheimer Report 2011: The benefits of early diagnosis and intervention https://www.alz.co.uk/research/WorldAlzheimerReport2011.pdf.

[8] Bamford C, Eccles M, Steen N, Robinson L (2007). Can primary care record review facilitate earlier diagnosis of dementia?. Fam Pract.

[9] Koch T, Iliffe S (2010). Rapid appraisal of barriers to the diagnosis and management of patients with dementia in primary care: a systematic review. BMC Fam Pract.

[10] Mittelman M, Haley W, Clay O, Roth D (2006). Improving caregiver well-being delays nursing home placement of patients with Alzheimer disease. Neurology.

[11] Derksen E, Vernooij-Dassen M, Gillissen F, Olde-Rikkert M, Scheltens P (2005). The impact of diagnostic disclosure in dementia: a qualitative case analysis. Int Psychogeriatr.

[12] de Vugt ME, Verhey FR (2013). The impact of early dementia diagnosis and intervention on informal caregivers. Prog Neurobiol.

[13] Zwingmann I, Hoffmann W, Michalowsky B, Dreier-Wolfgramm A, Hertel J, Wucherer D, et al. Supporting family dementia caregivers: testing the efficacy of dementia care management on multifaceted caregivers’ burden. Aging Ment Health. 2017;22(7):889-96. 10.1080/13607863.2017.1399341 29156941

[14] Foley T, Boyle S, Jennings A, Smithson WH (2017). “We're certainly not in our comfort zone”: a qualitative study of GPs’ dementia-care educational needs. BMC Fam Pract.

[15] Ahmad S, Orrell M, Iliffe S, Gracie A (2010). GPs’ attitudes, awareness, and practice regarding early diagnosis of dementia. Br J Gen Pract.

[16] Cahill S, Clark M, O'Connell H, Lawlor B, Coen RF, Walsh C (2008). The attitudes and practices of general practitioners regarding dementia diagnosis in Ireland. Int J Geriatr Psychiatry.

[17] Turner S, Iliffe S, Downs M, Wilcock J, Bryans M, Levin E (2004). General practitioners’ knowledge, confidence and attitudes in the diagnosis and management of dementia. Age Ageing.

[18] Low LF, McGrath M, Swaffer K, Brodaty H. Communicating a diagnosis of dementia: a systematic mixed studies review of attitudes and practices of health practitioners. Dementia. 2018;0(0):1–50. 10.1177/1471301218761911. 10.1177/1471301218761911 29544345

[19] Pentzek M, Abholz HH, Ostapczuk M, Altiner A, Wollny A, Fuchs A (2009). Dementia knowledge among general practitioners: first results and psychometric properties of a new instrument. Int Psychogeriatr.

[20] Thyrian JR, Hoffmann W (2012). Dementia care and general physicians--a survey on prevalence, means, attitudes and recommendations. Cent Eur J Public Health.

[21] Perry M, Draskovic I, Lucassen P, Vernooij-Dassen M, van Achterberg T, Rikkert MO (2011). Effects of educational interventions on primary dementia care: a systematic review. Int J Geriatr Psychiatry.

[22] Bamford C, Lamont S, Eccles M, Robinson L, May C, Bond J (2004). Disclosing a diagnosis of dementia: a systematic review. Int J Geriatr Psychiatry.

[23] Mason RL, Annear MJ, Lo A, McInerney F, Tierney LT, Robinson AL (2016). Development and preliminary psychometric properties of the general practitioner attitudes and confidence scale (GPACS-D) for dementia. BMC Fam Pract.

[24] Pentzek M, Fuchs A, Abholz HH (2005). Die Einstellungen der Hausärzte zu Demenzen. Nervenheilkunde.

[25] Petrazzuoli F, Vinker S, Koskela TH, Frese T, Buono N, Soler JK (2017). Exploring dementia management attitudes in primary care: a key informant survey to primary care physicians in 25 European countries. Int Psychogeriatr.

[26] Bradford A, Kunik ME, Schulz P, Williams SP, Singh H (2009). Missed and delayed diagnosis of dementia in primary care: prevalence and contributing factors. Alzheimer Dis Assoc Disord.

[27] Aminzadeh F, Molnar FJ, Dalziel WB, Ayotte D (2012). A review of barriers and enablers to diagnosis and management of persons with dementia in primary care. Can Geriatr J.

[28] Dubois B, Padovani A, Scheltens P, Rossi A, Dell'Agnello G (2016). Timely diagnosis for Alzheimer's disease: a literature review on benefits and challenges. J Alzheimers Dis.

[29] Mattsson N, Brax D, Zetterberg H. To know or not to know: ethical issues related to early diagnosis of Alzheimer's disease. Int J Alzheimers Dis. 2010;2010:1-4. 10.4061/2010/841941 PMC2925376 20798843

[30] Monsch AU, Bula C, Hermelink M, Kressig RW, Martensson B, Mosimann U (2013). Consensus 2012--diagnosis and treatment of patients with dementia in Switzerland. Rev Med Suisse.

[31] Giezendanner S, Monsch AU, Kressig RW, Mueller Y, Streit S, Essig S, et al. Early diagnosis and management of dementia in general practice - how do Swiss GPs meet the challenge? Manuscript accepted for publication at Swiss Med Wkly. 10.4414/smw.2018.14695 30576570

[32] Kim J-O, Mueller CW (1978). Factor analysis: statistical methods and practical issues: sage.

[33] R Development Core Team (2015). R: A language and environment for statistical computing.

[34] Subramaniam M, Ong HL, Abdin E, Chua BY, Shafie S, Siva Kumar FD (2018). General Practitioner's attitudes and confidence in managing patients with dementia in Singapore. Ann Acad Med Singap.

[35] Thyrian JR, Eichler T, Pooch A, Albuerne K, Dreier A, Michalowsky B (2016). Systematic, early identification of dementia and dementia care management are highly appreciated by general physicians in primary care - results within a cluster-randomized-controlled trial (DelpHi). J Multidiscip Healthc.

[36] Tang EY, Birdi R, Robinson L. Attitudes to diagnosis and management in dementia care: views of future general practitioners. Int Psychogeriatr. 2016;30(3):425-30. 10.1017/S1041610216001204 27502828

[37] Fox M, Fox C, Cruickshank W, Penhale B, Poland F, Steel N (2014). Understanding the dementia diagnosis gap in Norfolk and Suffolk: a survey of general practitioners. Qual Prim Care.

[38] Singer PA, Robertson G, Roy DJ (1996). Bioethics for clinicians: 6. Advance care planning. CMAJ.

[39] World health O (1990). Cancer pain relief and palliative care: report of a WHO expert committee [meeting held in Geneva from 3 to 10 July 1989].

[40] Sepulveda C, Marlin A, Yoshida T, Ullrich A (2002). Palliative Care: the World Health Organization's global perspective. J Pain Symptom Manage.

[41] Cahill S, Clark M, Walsh C, O'Connell H, Lawlor B (2006). Dementia in primary care: the first survey of Irish general practitioners. Int J Geriatr Psychiatry.

[42] Kaduszkiewicz H, Wiese B, van den Bussche H (2008). Self-reported competence, attitude and approach of physicians towards patients with dementia in ambulatory care: results of a postal survey. BMC Health Serv Res.

[43] Stephan A, Bieber A, Hopper L, Joyce R, Irving K, Zanetti O (2018). Barriers and facilitators to the access to and use of formal dementia care: findings of a focus group study with people with dementia, informal carers and health and social care professionals in eight European countries. BMC Geriatr.

[44] Michalowsky B, Kostev K, Hoffmann W, Bohlken J (2018). Z Gerontol Geriatr.

[45] Bohlken J, Michalowsky B, Kostev K (2017). Sharp increase in newly diagnosed patients with dementia in German primary care practices 2013. Better diagnostic process or monetary incentives?. Fortschr Neurol Psychiatr.

[46] Mason A, Liu D, Kasteridis P, Goddard M, Jacobs R, Wittenberg R (2018). Investigating the impact of primary care payments on underdiagnosis in dementia: a difference-in-differences analysis. Int J Geriatr Psychiatry.

[47] Otte IC, Jung C, Elger BS, Bally K (2014). Advance directives and the impact of timing. A qualitative study with Swiss general practitioners. Swiss Med Wkly.

[48] Sebo P, Maisonneuve H, Cerutti B, Fournier JP, Senn N, Haller DM (2017). Rates, delays, and completeness of general Practitioners’ responses to a postal versus web-based survey: a randomized trial. J Med Internet Res.

[49] Bonevski B, Magin P, Horton G, Foster M, Girgis A (2011). Response rates in GP surveys - trialling two recruitment strategies. Aust Fam Physician.

[50] Creavin ST, Creavin AL, Mallen CD (2011). Do GPs respond to postal questionnaire surveys? A comprehensive review of primary care literature. Fam Pract.

[51] Ryynanen OP, Myllykangas M, Kinnunen J, Halonen P, Takala J (2000). Prioritization attitudes among doctors and nurses examined by a scenario method. Int J Technol Assess Health Care.

[52] Fink HA, Jutkowitz E, McCarten JR, Hemmy LS, Butler M, Davila H (2018). Pharmacologic interventions to prevent cognitive decline, mild cognitive impairment, and clinical Alzheimer-type dementia: a systematic review. Ann Intern Med.

[53] Honig LS, Vellas B, Woodward M, Boada M, Bullock R, Borrie M (2018). Trial of Solanezumab for mild dementia due to Alzheimer's disease. N Engl J Med.

[54] Sacks CA, Avorn J, Kesselheim AS (2017). The failure of Solanezumab - how the FDA saved taxpayers billions. N Engl J Med.

