# Supplementary material for: General practitioners’ attitudes towards early diagnosis of dementia: a cross-sectional survey
Source: BMC Fam Pract. 2019 May 20;20:65. doi: 10.1186/s12875-019-0956-1 (PMC6528190; doi:10.1186/s12875-019-0956-1)
Supplement: Supplementary file 1 — Table S1. Stages of dementia at the point of first diagnosis used in the questionnaire. Table S2. Demographic, regional and professional characteristics of the respondent GPs. Table S3. Frequency of agreement with attitudes towards timely diagnosis (N = 882). Table S4. Frequency and summary statistics of measures taken after the diagnosis of mild dementia. Table S5. Supplementary information on exploratory factor analysis. Table S6. Figure: Ranked attitudes regarding dementia recognition and care. Attitudes were ranked according to GPs’ agreement. Table S7. Supplementary measures for the quality of the scales. Table S8. Summary table for subscales of attitudes of agreement with attitudes towards timely diagnosis. (DOCX 205 kb) [file 12875_2019_956_MOESM1_ESM.docx]

Additional file 1

S 1. Stages of dementia at the point of first diagnosis used in the questionnaire

Mild cognitive impairement (MCI): Abnormalities in cognitive testing but patient is able to compensate memory difficulties in everyday activities.

Mild dementia: Slight difficulties in the activities of daily living (e.g., finances, travelling) compared with before. The patient lives largely independently at home (MMSE 20-30).

Moderate dementia: Cognitive and everyday impairments with the patient being selectively or continuously dependent on help (e.g. making calls, taking medication, taking care of the clothes, organizing meals). Living at home is possible with support (MMSE of 10-19).

Severe dementia Cognition and the activities of daily living are severely impaired to such an extent that continuous care is necessary (e.g. food intake, personal hygiene, continence) (MMSE <10)”.

S 2. Demographic, regional and professional characteristics of the respondent GPs.

|  | Respondents (N = 882) | | Total sample (N = 4460) | Statistic (Chi-squared or t-test) | P value |
| --- | --- | --- | --- | --- | --- |
| Variable | N* (%) | | N* (%) |  |  |
| Gender (male) | 617 (70.0) | | 3077 (69) | 0.28 | 0.599 |
| Age (years) (Mean, SD) | 55.8 (8.86) | | 56.7 (8.7) | -2.77 | 0.006 |
| Language region |  |  |  |  |  |
| German | 692 (78.5) | | 3355(75.2) | 6.16 | 0.046 |
| French | 151 (17.1) | | 926 (20.8) |  |  |
| Italian | 39 (4.42) | | 179 (4.0) |  |  |
| Major Swiss regions |  |  |  |  |  |
| Lake Geneva (GE, VS, VD) | 106 (12.3) | |  |  |  |
| Middle Switzerland (BE, SO, FR, NE, JU) | 216 (25.1) | |  |  |  |
| Northwestern Switzerland (BS, BL, AG) | 161 (18.7) | |  |  |  |
| Eastern Switzerland (SG, TG, AI, AR, GL, SH, GR) | 130 (15.1) | |  |  |  |
| Ticino (TI) | 38 (4.41) | |  |  |  |
| Central Switzerland (UR, SZ, OW, NW, LU, ZG) | 72 (8.35) | |  |  |  |
| Zurich (ZH) | 140 (16.2) | |  |  |  |
| Type of area in which practice is located |  |  |  |  |  |
| City | 297 (34.0) | |  |  |  |
| Agglomeration of a city | 274 (31.4) | |  |  |  |
| Countryside | 302 (34.6) | |  |  |  |
| Years in practice (mean, SD) | 20.0 (9.92) | |  |  |  |
| Hours of work per week (patients and administration) (mean, SD) | 46.0 (14.2) | |  |  |  |
| Consultations per half day (mean, SD) | 12.94 (4.74) | |  |  |  |
| Estimated percentage of patients over the age of 70 (mean, SD) | 36.3 (17.5) | |  |  |  |

* if not otherwise specified, number of responders (N) and percentage of total (%) is reported. Missing values for age, n = 32 (3.63%), no. hours work per week, n = 4 (0.45%), no. consultations per half day, n = 9 (1.02%), type of area in which surgery is located, n = 9 (1.02%), estimate of the percentage of patients over the age of 70, n = 10 (1.13%), no. years in practice, n = 2 (0.23%)

S 3. Frequency of agreement with attitudes towards timely diagnosis (N = 882)

| Attitudes | Median | IQR | Agree (%) | Partially agree(%) | Disagree  (%) | Missing data N |
| --- | --- | --- | --- | --- | --- | --- |
| It is important to look actively for early signs of dementia | 4 | 3.5 - 4.5 | 473(54) | 234(27) | 170(19) | 5 (0.57) |
| The early recognition of dementia usually serves the welfare of the patient | 3 | 2.5 - 3.5 | 437(50) | 276(31) | 169(19) | 0 (0.00) |
| Providing a patient with a dementia diagnosis is providing a diagnosis that is not clinically actionable | 3 | 2 - 4 | 316(36) | 266(30) | 295(33) | 5 (0.57) |
| The early recognition of dementia usually serves the welfare of the patient’s relatives. | 4 | 3.5 - 4.5 | 538(61) | 245(28) | 95(11) | 4 (0.45) |
| The present treatment options with anti-dementia drugs usually have a positive influence on the course of the disease | 3 | 2.5 - 3.5 | 157(18) | 324(37) | 397(45) | 4 (0.45) |
| Managing dementia is more often frustrating than rewarding | 2 | 1.5 - 2.5 | 158(18) | 250(28) | 470(54) | 4 (0.45) |
| Enablers of timely diagnosis |  |  |  |  |  |  |
| With a timely diagnosis GPs/patients may take actions to improve disease outcome. | 3 | 2.5 - 3.5 | 429(48) | 244(28) | 205(23) | 4 (0.45) |
| A timely diagnosis may delay institutionalization | 4 | 3.5 - 4.5 | 471(54) | 253(29) | 155(18) | 3 (0.34) |
| With a timely diagnosis dangerous and difficult situations can be reduced | 4 | 3.5 - 4.5 | 733(83) | 113(13) | 32(3) | 4 (0.45) |
| A timely diagnosis may enable planning for the future, organize support and care | 4 | 3.5 - 4.5 | 726(82) | 120(14) | 32(3) | 4 (0.45) |
| A timely diagnosis may minimize the strain and insecurity of patients and their informal family caregivers | 4 | 3.5 - 4.5 | 699(80) | 148(17) | 29(3) | 6 (0.68) |
| With a timely diagnosis patients may make appropriate legal arrangements | 4 | 3.5 - 4.5 | 686(79) | 166(19) | 22(3) | 8 (0.91) |
| Barriers to timely diagnosis |  |  |  |  |  |  |
| Patients with dementia can be a drain on resources that should be used for late stage dementia patients | 2 | 1.5 - 2.5 | 113(13) | 182(21) | 575(66) | 12 (1.36) |
| Concern about possible burden or stigmatization of patients with diagnosis | 2 | 1.5 - 2.5 | 144(16) | 203(23) | 531(60) | 4 (0.45) |
| Embarrassment or discomfort in disclosing the diagnosis to the patient or family | 2 | 1.5 - 2.5 | 82(10) | 109(12) | 684(78) | 7 (0.79) |
| Time constraints in carrying out the necessary procedures to diagnose dementia | 2 | 1.5 - 2.5 | 174(20) | 199(23) | 503(58) | 6 (0.68) |
| Inadequate financial remuneration hinders diagnosis | 4 | 3 - 5 | 452(53) | 150(18) | 255(30) | 25 (2.83) |
| A timely diagnosis may increase suicide risk | 2 | 1.5 - 2.5 | 120(14) | 231(26) | 523(59) | 8 (0.91) |
| Patients or families do not feel so much psychological strain as to need a diagnosis | 2 | 1.5 - 2.5 | 136(18) | 199(26) | 433(56) | 114 (12.93) |
| A timely diagnosis is linked to a lot of paperwork | 3 | 2.5 - 3.5 | 209(24) | 270(31) | 388(44) | 15 (1.70) |

5-point Likert scale: 1 = strongly disagree, 2= disagree, 3 = partially agree, 4 = agree, 5 = strongly agree. To summarize the responses, scores from Likert scales were partly grouped to rank the degree of agreement (1 + 2 = “do not agree”; 3 = “partially agree”; 4 + 5 = “agree”).

S 4. Frequency and summary statistics of measures taken after the diagnosis of mild dementia

| Strategies | Frequent, n (%) | 50% of cases  n (%) | Not frequent, n (%) | Mdn | IQR |
| --- | --- | --- | --- | --- | --- |
| Counselling of relatives | 613(71) | 159(18) | 92(11) | 75 | 50 - 100 |
| Assessment of driving aptitude | 555(66) | 131(16) | 153(18) | 75 | 50 - 100 |
| Measures to minimise cardiovascular risks | 528(63) | 156(19) | 149(18) | 75 | 50 - 100 |
| Prepare advance directives or to designate a power of attorney | 386(46) | 202(24) | 259(31) | 50 | 25 - 75 |
| Memory training activities | 351(42) | 166(20) | 321(38) | 50 | 25 - 75 |
| Prescription of ginkgo biloba | 308(36) | 163(19) | 381(45) | 50 | 25 - 75 |
| Refer patient to counselling centre, e.g. Alzheimer’s association | 296(35) | 226(27) | 325(38) | 50 | 25 - 75 |
| Pharmacological monotherapy (acetylcholinesterase inhibitors or memantine) | 235(29) | 209(25) | 388(47) | 50 | 25 - 75 |
| Other non-pharmacological treatments (involving music, painting, dancing, coaching strategies or conversations to cope with the situation) | 174(21) | 147(18) | 510(61) | 25 | 0 - 50 |
| Wait-and-see strategy with no intervention | 161(21) | 167(21) | 464(58) | 25 | 25- 50 |
| Home visits to assess specific living conditions | 91(12) | 124(17) | 526(71) | 25 | 0 - 50 |
| Pharmacological combination therapy (acetylcholinesterase inhibitors and memantine) | 21(3) | 41(5) | 710(92) | 0 | 0 - 25 |

AChEI: acetylcholinesterase inhibitor, AD: Alzheimer’s disease

5-point Likert scale: 1 = 0% (never), 2=25%, 3 =50%, 4 = 75%, 5 = 100% (always). To summarize the responses, the scores from Likert scales were partly grouped to rank the degree of the frequency (1+2 = “infrequent”, 3 = “50% of cases”, 4 + 5 = “frequent”)

S 5. Supplementary information on exploratory factor analysis

The exploratory factor analysis was performed with the function “fa” from the “psych” package in R using the polychoric correlation matrix as input, the number of factors = 2, the “Ordinary Least Squared/Minres” factoring and an orthogonal rotation “varimax” as we were interested in two uncorrelated factors. Negatively loaded items were reverse scored to establish a total congruent score. We created summary scores by computing unweighted averages of items loading >0.30 on each factor. Scores ranged from 1 to 5, with greater scores representing greater agreement with the attitude.

S 6. Figure: Ranked attitudes regarding dementia recognition and care. Attitudes were ranked according to GPs’ agreement.


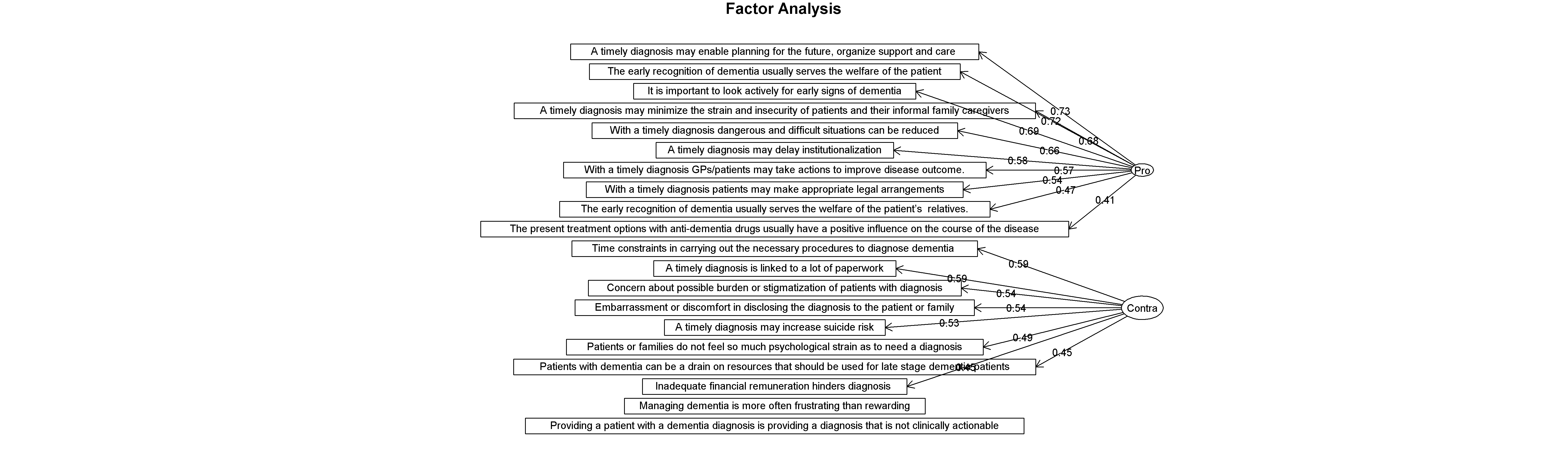
 Factor structures resulting from exploratory factor analysis. The arrows represent the factor loadings and come from the latent variables (in circles) to the observed variables (in rectangles). N =  882. This figure was created using the R package “psych”.

S 7. Supplementary measures for the quality of the scales

To validate the model, the root mean square of residuals and root mean square error of approximation index, which should both be close to zero, were considered. Internal consistency measures of reliability were estimated by calculating Cronbach’s alpha [53] and McDonald’s omega estimates of general and total factor saturation [54, 55]. Further measures of the quality of the scales were assessed, such as Guttman’s lambda 6, to consider the amount of variance in each item that can be accounted for the linear regression of all of the other items and signal/noise ratio. Convergent and discriminant validity of the subscales of the factor solution were checked using the function mtmm of the “psy” package in R. Convergent validity is a type of construct validity which is a measure of whether the same constructs are related to each other. Conversely, discriminant validity measures whether the constructs are unrelated to each other. We generally consider that a correlation of 0.3 or above is indicative of a “meaningful” correlation for two items belonging to the same subscale. Discriminant validity exists if the correlation between an item and its factor is higher than its correlation with other factors.

S 8. Summary table for subscales of attitudes of agreement with attitudes towards timely diagnosis

|  | Factor 1  Enablers of early dementia diagnosis | Factor 2  Barriers to early dementia diagnosis |
| --- | --- | --- |
| SS loadings | 4.38 | 2.44 |
| Proportion variance | 0.22 | 0.12 |
| Cumulative variance | 0.22 | 0.34 |
| Proportion explained | 0.64 | 0.36 |
| Cumulative proportion | 0.64 | 1.00 |
| Eigen value | 5.32 | 2.77 |
| Omega general | 0.59 | 0.42 |
| Omega total | 0.88 | 0.79 |
| Alpha (based upon the covariances) | 0.82 | 0.70 |
| Guttman’s lambda 6 reliability | 0.85 | 0.71 |
| Homogeneity (average interitem correlation) | 0.32 | 0.23 |
| Signal/noise ratio | 4.69 | 2.43 |
| Convergent validity^a^ | 0.47 - 0.72 | 0.5 - 0.63 |
| Divergent validity | 0.02 - 0.36 | 0 - 0.23 |
| Summary statistics of the averaged total score |  |  |
| No. of items | 10.00 | 8.00 |
| Number of valid cases | 853.00 | 733.00 |
| Mean | 3.66 | 2.46 |
| Standard deviation | 0.58 | 0.61 |
| Median | 3.70 | 2.50 |
| trimmed mean (with trim defaulting to .1) | 3.67 | 2.46 |
| mad: median absolute deviation (from the median) | 0.59 | 0.56 |
| Minimum | 1.70 | 1.00 |
| Maximum | 5.00 | 4.13 |
| Range | 3.30 | 3.13 |
| Skew | -0.21 | 0.03 |
| Kurtosis | -0.13 | -0.20 |
| standard error | 0.02 | 0.02 |

Omega hierarchical is the amount of the total test variance associated with the general factor.

^a^ based on Spearman correlations. The validity of the model was acceptable with RMSEA index = 0.12 and RMSR = 0.07), Tucker-Lewis Index = 0.618. The correlation of an item with the global score of its subscale was above its correlations with the global score of the other subscales (see convergent validity and divergent validity).
